# Beliefs on social distancing and face mask practices during the COVID-19 pandemic in low- and middle-income countries: a cross-sectional study

**DOI:** 10.12688/f1000research.79534.1

**Published:** 2022-02-17

**Authors:** Harapan Harapan, Amanda Yufika, Samsul Anwar, Youdiil Ophinni, Chika Yamada, Khan Sharun, Mahir Gachabayov, Marhami Fahriani, Milda Husnah, Rawan Raad, Namareg ME. Khiri, Rashed YA Abdalla, Wajdi Kacem, Zeineb Teyeb, Khaoula Aloui, Manel Ferjani, Dalia A. Deeb, Dina Emad, Kirellos S Abbas, Suhrud Panchawagh, Sunil Anandu, Md Ariful Haque, Lirane ED. Ferreto, María FC. Briones, Rocío BI. Morales, Sebastián Lazcano-Díaz, Abiodun Durosinmi, Esther N. Adejumo, Elham Babadi, Edris Kakemam, Irfan Ullah, Najma I. Malik, Francesco Rosiello, Talha B. Emran, Firzan Nainu, Guilherme W. Wendt, Morteza Arab-Zozani, Abram L. Wagner, Mudatsir Mudatsir

**Affiliations:** 1 Medical Research Unit, School of Medicine, Universitas Syiah Kuala, Banda Aceh, 23111, Indonesia; 2 Tropical Disease Centre, School of Medicine, Universitas Syiah Kuala, Banda Aceh, 23111, Indonesia; 3 Department of Microbiology, School of Medicine, Universitas Syiah Kuala, Banda Aceh, 23111, Indonesia; 4 Department of Family Medicine, School of Medicine, Universitas Syiah Kuala, Banda Aceh, 23111, Indonesia; 5 Department of Statistics, Faculty of Mathematics and Natural Sciences, Universitas Syiah Kuala, Banda Aceh, 23111, Indonesia; 6 Ragon Institute of MGH, MIT and Harvard, Cambridge, 02139, USA; 7 Laboratory of Host Defense, WPI Immunology Frontier Research Center (IFReC), Osaka University, Osaka, 565-0874, Japan; 8 Department of Environmental Coexistence, Center for Southeast Asian Studies, Kyoto University, Kyoto, 606-8304, Japan; 9 Division of Surgery, ICAR-Indian Veterinary Research Institute, Izatnagar, Bareilly, 243122, India; 10 Department of Abdominal Surgery, Vladimir City Emergency Hospital, Vladimir, 600014, Russian Federation; 11 Master Program of Biology, Faculty of Mathematics and Natural Sciences, Universitas Syiah Kuala, Banda Aceh, 23111, Indonesia; 12 Faculty of Medicine and General Surgery, Sudan University of Science and Technology, Khartoum, 407, Sudan; 13 Faculty of Medicine, University of Khartoum, Omdurman, 11111, Sudan; 14 Faculty of Medicine, University of Bahri, Khartoum, 11111, Sudan; 15 Department of Emergency Medicine, Faculty of Medicine of Tunis, University Tunis el Manar, Tunis, 2074, Tunisia; 16 Department of Internal Medicine, Faculty of Medicine of Tunis, University of Tunis El Manar, Tunis, 2074, Tunisia; 17 Faculty of Dental Medicine Monastir, University of Tunis El Manar, Monastir, 5000, Tunisia; 18 Faculty of Medicine, Zagazig University, El-sharkia, 44519, Egypt; 19 Faculty of Medicine, Ain Shams University Nasr City, Cairo, 1181, Egypt; 20 Faculty of Medicine, Alexandria University, Alexandria, 21131, Egypt; 21 Department of General Medicine, Smt. Kashibai Navale Medical College and General Hospital, Pune, 516599001, India; 22 Division of Veterinary Parasitology, ICAR-Indian Veterinary Research Institute, Izatnagar, Bareilly, Uttar Pradesh, 243122, India; 23 Department of Orthopedic Surgery, Yanan Hospital Affiliated to Kunming Medical University, Kunming, Yunnan, 650000, China; 24 Department of Public Health and Postgraduate Program in Applied Health Sciences, Faculty of Medicine, Western Paraná State University, Francisco Beltrão, 85601-970, Brazil; 25 Faculty of Medicine, University of La Frontera, Temuco, 4781218, Chile; 26 Covenant University Medical Center, Ijebu Ode, Ogun, 120101, Nigeria; 27 Department of Medical Laboratory Science, Babcock University, Ilishan-Remo, Ogun State, 121103, Nigeria; 28 Research Fellow, Mayo Clinic, Rochester, 14604, USA; 29 Tabriz Health Services Management Research Center, Tabriz University of Medical Sciences, Tabriz, 516599001, Iran; 30 Department of Internal Medicine, Kabir Medical College, Gandhara University, Peshawar, 25000, Pakistan; 31 Department of Psychology, University of Sargodha, Sargodha, 40100, Pakistan; 32 Department of Public Health and Infectious Disease, Sapienza-University of Rome, Rome, 00185, Italy; 33 Department of Pharmacy, BGC Trust University Bangladesh, Chittagong, 4381, Bangladesh; 34 Department of Pharmacy, Faculty of Pharmacy, Hasanuddin University, Tamalanrea, Makassar, 90245, Indonesia; 35 Department of Life Sciences, Faculty of Medicine, Western Paraná State University, Francisco Beltrão, 85601-970, Brazil; 36 Social Determinants of Health Research Center, Birjand University of Medical Sciences, Birjand, 97, Iran; 37 Department of Epidemiology, University of Michigan, Ann Arbor, MI, 48109, USA

**Keywords:** COVID-19, face mask, social distancing, preventive measure, practice

## Abstract

Introduction: Social distancing and wearing a face mask are highly recommended to mitigate the transmission of coronavirus disease 2019 (COVID-19). However, the success of these strategies relies on individuals’ adherence and public compliance. This study was conducted to assess the level of belief in social distancing and face mask practices in communities in low- and middle-income countries (LMICs) and to identify their possible determinants.

Methods: A cross-sectional study was conducted in ten LMICs countries in Asia, Africa, and South America from February to May 2021. A questionnaire was used to assess the belief, practice, and their plausible determinants. Identification of the associated determinants was performed using a logistic regression model.

Results: Our data revealed that only 62.6% and 66.9% of the participants had good beliefs in social distancing and good face mask practices, respectively. Residing in the Americas, having a healthcare-related job, knowing people in immediate social environment who are or have been infected and exposure to information of COVID-19 cases on social media or TV were factors significantly associated with good belief in social distancing. Residing country, gender, monthly household income, type of job and exposure to information of COVID-19 cases were significantly associated with face mask wearing practice.

Conclusion: The proportion of participants having good beliefs in social distancing and good face mask practices is relatively low (<75%). Hence, sustained health campaigns regarding social distancing benefits and face mask-wearing practices during COVID-19 are critical in LMICs.

## Introduction

The coronavirus disease 2019 (COVID-19) pandemic is continuing to affect millions of people globally following the initial emergence of severe acute respiratory syndrome coronavirus 2 (SARS-CoV-2) in Wuhan, China. ^1^^,^^2^ The COVID-19 pandemic has massively impacted existing health care systems worldwide and in particular in low- and middle-income countries (LMICs). ^3^^,^^4^ A syndemic of COVID-19 and other endemic infections could be particularly burdensome to tropical countries. ^5^^,^^6^ In the present scenario, strict adherence to social distancing (maintaining a physical distance of at least 2 meters), wearing a face mask, and regular handwashing are essential practices that are highly recommended for preventing human-to-human transmission of SARS-CoV-2. ^7^^,^^8^

Social distancing plays a critical role in slowing the rapid transmission of SARS-CoV-2 within the community. ^9^^,^^10^ Government-imposed social distancing measures have been found to be associated with a significant reduction in the cumulative incidence of COVID-19 worldwide. ^11^ Furthermore, people living in communities with better social distancing practices had a lower predicted risk of COVID-19 than those living in poor social distancing situations. ^12^ Accordingly, strict social distancing policies – prohibiting large gatherings and close social interactions between individuals from different households – could help mitigate the spread of SARS-CoV-2. ^13^ Achieving a high degree of compliance in social distancing behaviors is essential for preventing the spread of disease within the community. ^10^ Therefore, public health campaigns have to be conducted to improve the acceptability and adherence to the social distancing policies within the community. ^13^^,^^14^ Similarly to social distancing, mask-wearing will help slow down the spread of SARS-CoV-2, which is essential to allow health care facilities to continue functioning. ^10^ Wearing masks could also reduce SARS-CoV-2 transmission even in settings of poor social distancing. ^12^

Studies from European countries indicate that mandatory stay-at-home orders directly impacted population mobility and subsequently decreased the COVID-19 case growth rate during the pandemic. ^9^ Compliance of the public to the directives given by the governments and public health agencies will decide the effectiveness of these directives. Therefore, understanding beliefs in social distancing benefits and face mask practices among community members will help us formulate targeted public health campaigns that focus on specific subgroups to improve their acceptability and adherence to various prosocial behaviors.

This study sought to determine: (a) the level of beliefs in social distancing benefits and face mask practices; and (b) the possible determinants associated with belief in social distancing benefits and face mask practices among community members in ten LMICs in Asia, Africa, and South America.

## Methods

### Study design and setting

We conducted a cross-sectional study among community members in Asia (Bangladesh, India, Iran and Pakistan), Africa (Egypt, Nigeria, Sudan and Tunisia) and South America (Brazil and Chile) from February to May 2021. Community members were defined as all residents who have lived in one of these areas for at least three months prior to the study.

### Study population and sampling

Adults aged over 18 years old living in one of the studied countries and able to respond each question in the survey were considered eligible for the study. Illiterate individuals or those who needed help to complete the survey were considered ineligible. To recruit the minimal sample size, we sought a 5% margin of error, 95% confidence level and a 50% conservative estimate of respondents having good beliefs in social distancing benefits and face mask practices. The samples were recruited using a non-probability sampling method, convenience sampling approach.

### Data collection

The platform SurveyMonkey was used to host the anonymous online survey and the links to the survey were distributed on social media platforms such as Twitter, Facebook, and WhatsApp. A copy of the survey can be found under *Extended data.* ^37^ The invitation to participate in the survey was posted on Twitter and Facebook and shared on WhatsApp and the invited individuals were requested to share the invitation to their phone contacts. The survey consisted of an introduction page where information on the study was provided; an informed consent page where respondents had to provide consent to participate; and the main survey where respondents answered questions about their demographic background and their beliefs regarding social distancing and face mask practices. Approximately 15 minutes was required to complete all the questions. The questions within the questionnaire were tested and validity was confirmed prior to being used in the study.

### Study variables

***Response variables***

There are two response variables of the study: belief in the benefits of social distancing and face mask practices during the pandemic. To assess belief in social distancing benefits, the participants were asked to respond to three statements: (1) “Social distancing can protect yourself from COVID-19”; (2) “Social distancing can protect your child or children from COVID-19”; and (3) “Social distancing can protect your parents from COVID-19”. The possible responses and the scores were: “Strongly agree (score 5)”, “Agree (4)”, “Neither agree nor disagree (3)”, “Disagree (2)”, and “Strongly disagree (1)”. For each participant, the score from each statement was summed and the total score ranged from 3 to 15, in which the higher score indicates better belief in the benefits of social distancing. Those with a score of more than 80% (i.e., 13 or more) were classified as having a good belief in social distancing benefits while those with a score of less than 80% (i.e., 12 or less) were classified as having a poor belief. The 80% cut-off has been used to dichotomize the variables into good and poor previously. ^15^^,^^16^

The respondents were asked two questions to assess the face mask practices during the pandemic. These questions were about whether the respondents would: (1) wear a mask at work/school; and (2) wear a mask at the grocery store or other food vendors. The possible responses and the given score were: “Yes, during whole time (score 2)”, “Yes, for part of the time (score 1)”, “No (score 0)”, and “Not applicable (not going out for a whole week)”. The total score ranged between 0 to 4 and those wearing face masks during the whole time in both activities (score 4) were classified as having a good practice of face mask use, whereas those who did not (i.e., the score was less than 4) were classified as having poor practice. All participants who stated “not applicable" for one of the questions (they did not go to school/work or to a grocery store or other food vendor for the whole week) were excluded.

***Explanatory variables***

Several explanatory variables were included such as age, gender, urbanicity, monthly household income in USD, religion, occupation sector (healthcare- and non-healthcare-related), type of occupation, and the presence of COVID-19 comorbidities based on self-report, such as hypertension, diabetes, heart disease and pulmonary disease. The respondents were also asked whether they knew any people in their immediate social environment who are or have been infected with SARS-CoV-2. Respondents were also questioned about their exposure to information about individuals infected with the SARS-CoV-2 on TV or social media.

The perceived risk towards COVID-19 was also assessed for all respondents using two questions: (1) “What do you think are the chances that you will get COVID-19 in the next month?” and (2) “What do you think is your risk of dying from COVID-19 if infected? Response were assessed on a continuous scale of 0% to 100%. For each question, the respondents were classified into two: those with a perceived risk score equal to or less than 50% and those who had a score of more than 50%. In addition, the scores from both questions were summed and those who achieved more than 50% of the total score (i.e., 100 scores out of 200) were classified as having high perceived risk; conversely, those with less than 50% were classified as having a low perceived risk.

### Statistical analysis

A logistic regression was used to identify the explanatory variables associated with belief in benefits of social distancing and face mask practice in respondents. The analyses were conducted into two steps. First, in unadjusted logistic regressions, the associations of all explanatory variables with belief in benefits of social distancing and face mask practice were calculated separately. In this step, the crude odds ratio (OR) and 95% confidence interval (95%CI) of each explanatory variable for a good social distancing belief or good face mask practice were calculated. In the second step, those variables with p-values less than 0.25 at unadjusted logistic regression step were included in adjusted logistic regressions to calculate adjusted OR (aOR) and the 95% CI. The significance of OR and aOR were assessed at α=0.05. All analyses were conducted using SPSS version 24 (SPSS Inc., Chicago, IL, USA).

### Ethics and consent

This study was approved by Institutional Review Board of Universitas Syiah Kuala & Zainoel Abidin Hospital (approval number: 129/EA/FK-RSUDZA/2021) and Indonesian National Health Research and Development Ethics Commission (#1171012P). An introduction page consisting of information about the objectives, the benefits, and risks of the study was provided. All respondents provided consent by ticking a box before the survey could be opened. The survey account could only be accessed by the principal investigator to ensure the anonymity and confidentiality of participants.

## Results

### Demographic characteristics

During the study, 1,849 responses were received. In this present study we excluded 203 respondents due to incomplete data. A total of 1,646 respondents were included in the analysis of belief in social distancing benefits, where more than 20% of them were from India. The characteristics of the respondents are presented in Table 1. The full dataset can be found in the *Underlying data*. ^37^ Approximately 56% of the respondents were aged between 21-30 years old, and 58% were female. Out of the total, more than a third of the respondents earned less than USD500 monthly. During the time of this study, 1,139 (69.2%) of the respondents reported to know people in their immediate social environment who were or had been infected with SARS-CoV-2 and 352 (30.9%) of them reported that these individuals had serious presentations of illness. In addition, 1,525 (92.6%) had seen or read about individuals infected with SARS-CoV-2 on social media or TV of which 48.7% (802 out 1,525) believed that the cases were very serious. In total, 36.4% and 22.5% of the respondents believed that they had more than a 50% risk of getting COVID-19 and dying from COVID-19 if infected, respectively ( Table 1).

**Table 1.** Participants’ characteristics included in the assessment of determinants associated with belief in social distancing ( *n*=1646) and face-mask practice ( *n*=1306).

Variable	Belief in social distancing ( *n*=1646)	Face-mask practice (n=1306)
	*n* (%)	*n* (%)
Country		
Pakistan	262 (15.9)	181 (13.9)
Brazil	107 (6.5)	62 (4.7)
Chile	106 (6.4)	44 (3.4)
Egypt	98 (6.0)	88 (6.7)
India	337 (20.5)	302 (23.1)
Iran	141 (8.6)	121 (9.3)
Nigeria	161 (9.8)	142 (10.9)
Bangladesh	131 (8.0)	107 (8.2)
Sudan	174 (10.6)	141 (10.8)
Tunisia	129 (7.8)	118 (9.0)
Age group (year)		
<20	279 (17.0)	185 (14.2)
21-30	926 (56.3)	744 (57.0)
31-40	268 (16.3)	231 (17.7)
41-50	119 (7.2)	103 (7.9)
>51	54 (3.3)	43 (3.3)
Gender		
Male	691 (42.0)	570 (43.6)
Female	955 (58.0)	736 (56.4)
Urbanicity		
Rural	314 (19.1)	254 (19.4)
Urban	1332 (80.9)	1052 (80.6)
Monthly household income (USD)		
<500	616 (37.4)	495 (37.9)
500-999	289 (17.6)	225 (17.2)
500-999	192 (11.7)	151 (11.6)
2,000-2,999	148 (9.0)	108 (8.3)
3,000-4,999	127 (7.7)	96 (7.4)
5,000-7,999	100 (6.1)	75 (5.7)
≥8,000	174 (10.6)	156 (11.9)
Religion		
Islam	914 (55.5)	745 (57.0)
Christian/Protestant/Methodist/Lutheran/Baptist	178 (10.8)	151 (11.6)
Catholic	127 (7.7)	87 (6.7)
Hindu	238 (14.5)	211 (16.2)
Atheist or agnostic	87 (5.3)	43 (3.3)
Others	102 (6.2)	69 (5.3)
Healthcare-related job		
No	907 (55.1)	662 (50.7)
Yes	739 (44.9)	644 (49.3)
Occupation		
Self-employed	155 (9.4)	138 (10.6)
Employed for wages	415 (25.2)	360 (27.6)
Out of work for less or more than 1 year	73 (4.4)	50 (3.8)
Homemaker	34 (2.1)	24 (1.8)
Student	947 (57.5)	716 (54.8)
Retired or unable to work	22 (1.3)	18 (1.4)
Have hypertension		
No ^a^	1099 (66.8)	893 (68.4)
Yes ^b^	97 (5.9)	80 (6.1)
Do not know	450 (27.3)	333 (25.5)
Have diabetes		
No ^a^	1188 (72.2)	962 (73.7)
Yes ^b^	58 (3.5)	50 (3.8)
Do not know	400 (24.3)	294 (22.5)
Have heart disease		
No ^a^	1090 (66.2)	883 (67.6)
Yes ^b^	55 (3.3)	47 (3.6)
Do not know	501 (30.4)	376 (28.8)
Have pulmonary disease		
No ^a^	1041 (63.2)	845 (64.7)
Yes ^b^	90 (5.5)	76 (5.8)
Do not know	515 (31.3)	385 (29.5)
Know people in immediate social environment who are or have been infected with COVID-19		
No	507 (30.8)	366 (28.0)
Yes	1139 (69.2)	940 (72.0)
Have you seen or read about individuals infected with the COVID-19 on social media or TV?		
No	121 (7.4)	101 (7.7)
Yes	1525 (92.6)	1205 (92.3)
What do you think are the chances that you will get COVID-19 in the next month?		
<50%	1047 (63.6)	800 (61.3)
≥50%	599 (36.4)	506 (38.7)
What do you think is your risk of dying from COVID-19 if infected?		
<50%	1276 (77.5)	1017 (77.9)
≥50%	370 (22.5)	289 (22.1)

^a^ Have been tested or examined by a doctor but negative.

^b^ Have been diagnosed by a doctor.

There were 340 respondents who did not go to school/work or did not go out to a grocery store or other food vendors for the week prior to the study and therefore were excluded. A total of 1,306 respondents were included in the analysis to assess the factors associated with face mask use practices, whose characteristics are similar to the total respondents ( Table 1).

### Belief in social distancing benefits and associated determinants

In total, 1,031 (62.6%) respondents had a good belief in social distancing benefits during the COVID-19 pandemic. The numbers were varied significantly among countries; the highest percentage was in Chile (91.5%), while the lowest was in Pakistan (50.4%). In general, countries in South America such as Chile and Brazil had a higher proportion of respondents who believed in social distancing benefits compared to those in African and Asian countries. In the univariate analysis, country, urbanicity, religion, type of job, having a pulmonary disease, knowing people in the immediate social environment who are or have been infected with SARS-CoV-2, and having seen or read about individuals infected with the SARS-CoV-2 on social media or TV were all factors associated with the belief in benefits of social distancing in some degree ( Table 2).

**Table 2.** Unadjusted and adjusted logistic regression analyses showing factors associated with good belief in social distancing benefits ( *n*=1646).

Variable	Good belief *n* (%) - 1031	Unadjusted		Adjusted	
		OR (95% CI)	*p*–value	OR (95% CI)	*p*–value
Country					
Pakistan *(R)*	132 (50.4)	1		1	
Brazil	88 (82.2)	4.56 (2.63-7.92)	<0.001 **	3.01 (1.45-6.25)	0.003 *
Chile	97 (91.5)	10.61 (5.14-21.91)	<0.001 **	8.33 (3.55-19.54)	<0.001 **
Egypt	60 (61.2)	1.56 (0.97-2.50)	0.067	1.07 (0.63-1.82)	0.793
India	193 (57.3)	1.32 (0.95-1.83)	0.093	0.69 (0.40-1.20)	0.190
Iran	88 (62.4)	1.64 (1.08-2.48)	0.021 *	1.13 (0.68-1.89)	0.633
Nigeria	98 (60.9)	1.53 (1.03-2.28)	0.036 *	0.95 (0.52-1.72)	0.865
Bangladesh	78 (59.5)	1.45 (0.95-2.22)	0.087	1.06 (0.66-1.71)	0.796
Sudan	119 (68.4)	2.13 (1.43-3.18)	<0.001 **	1.40 (0.87-2.25)	0.167
Tunisia	78 (60.5)	1.51 (0.98-2.31)	0.061	1.00 (0.60-1.69)	0.992
Age group (year)					
<20 *(R)*	161 (57.7)	1		1	
21-30	587 (63.4)	1.27 (0.97-1.67)	0.087	1.13 (0.81-1.58)	0.483
31-40	166 (61.9)	1.19 (0.85-1.68)	0.313	0.98 (0.61-1.58)	0.920
41-50	78 (65.5)	1.39 (0.89-2.18)	0.145	1.39 (0.78-2.47)	0.264
>51	39 (72.2)	1.91 (1.00-3.62)	0.049 *	1.65 (0.76-3.60)	0.209
Gender					
Male *(R)*	428 (61.9)	1			
Female	603 (63.1)	1.05 (0.86-1.29)	0.619		
Urbanicity					
Rural *(R)*	171 (54.5)	1		1	
Urban	860 (64.6)	1.52 (1.19-1.95)	0.001 *	1.12 (0.85-1.48)	0.404
Monthly household income (USD)					
<500 *(R)*	394 (64.0)	1			
500-999	178 (61.6)	0.90 (0.68-1.21)	0.491		
500-999	123 (64.1)	1.00 (0.72-1.41)	0.980		
2,000-2,999	95 (64.2)	1.01 (0.70-1.47)	0.959		
3,000-4,999	79 (62.2)	0.93 (0.63-1.38)	0.708		
5,000-7,999	59 (59.0)	0.81 (0.53-1.25)	0.340		
≥8,000	103 (59.2)	0.82 (0.58-1.15)	0.251		
Religion					
Islam *(R)*	538 (58.9)	1		1	
Christian/Protestant/Methodist/Lutheran/Baptist	111 (62.4)	1.16 (0.83-1.61)	0.385	1.05 (0.64-1.73)	0.850
Catholic	100 (78.7)	2.59 (1.66-4.04)	<0.001 *	1.22 (0.66-2.27)	0.527
Hindu	149 (62.6)	1.17 (0.87-1.57)	0.295	1.60 (0.97-2.63)	0.064
Atheist or agnostic	64 (73.6)	1.95 (1.19-3.19)	0.008 *	0.88 (0.47-1.65)	0.698
Others	69 (67.6)	1.46 (0.95-2.26)	0.088	0.92 (0.54-1.58)	0.763
Healthcare-related job					
No *(R)*	540 (59.5)	1		1	
Yes	491 (66.4)	1.35 (1.10-1.65)	0.004 *	1.39 (1.10-1.77)	0.007 *
Occupation					
Self-employed *(R)*	89 (57.4)	1		1	
Employed for wages	284 (68.4)	1.61 (1.10-2.35)	0.014 *	1.49 (0.99-2.24)	0.058
Out of work for less or more than 1 year	43(58.9)	1.06 (0.60-1.87)	0.832	1.01 (0.56-1.84)	0.976
Homemaker	17 (50.0)	0.74 (0.35-1.56)	0.431	0.77 (0.34-1.71)	0.519
Student	586 (61.9)	1.20 (0.85-1.70)	0.291	1.23 (0.82-1.85)	0.327
Retired or unable to work	12 (54.5)	0.89 (0.36-2.18)	0.799	0.80 (0.31-2.11)	0.657
Have hypertension					
No ^a^ *(R)*	708 (64.4)	1		1	
Yes ^b^	55 (56.7)	0.72 (0.48-1.10)	0.131	0.80 (0.48-1.32)	0.377
Do not know	268 (59.6)	0.81 (0.65-1.02)	0.072	1.09 (0.78-1.52)	0.615
Have diabetes					
No ^a^ *(R)*	759 (63.9)	1		1	
Yes ^b^	36 (62.1)	0.93 (0.54-1.59)	0.778	1.45 (0.75-2.80)	0.269
Do not know	236 (59.0)	0.81 (0.65-1.03)	0.081	1.01 (0.71-1.43)	0.965
Have heart disease					
No ^a^ *(R)*	699 (64.1)	1		1	
Yes ^b^	30 (54.5)	0.67 (0.39-1.16)	0.152	0.75 (0.39-1.46)	0.394
Do not know	302 (60.3)	0.85 (0.68-1.06)	0.140	1.13 (0.77-1.67)	0.523
Have pulmonary disease					
No ^a^ *(R)*	674 (64.7)	1		1	
Yes ^b^	55 (61.1)	0.86 (0.55-1.33)	0.490	0.87 (0.52-1.48)	0.615
Do not know	302 (58.6)	0.77 (0.62-0.96)	0.019 *	0.81 (0.55-1.19)	0.282
Know people in immediate social environment who are or have been infected with COVID-19					
No *(R)*	284 (56.0)	1		1	
Yes	747 (65.6)	1.50 (1.21-1.85)	<0.001 **	1.41 (1.02-1.96)	0.038 *
Have you seen or read about individuals infected with the COVID-19 on social media or TV?					
No *(R)*	53 (43.8)	1		1	
Yes	978 (64.1)	2.29 (1.58-3.34)	<0.001 **	2.43 (1.60-3.69)	<0.001 **
What do you think are the chances that you will get COVID-19 in the next month?					
<50% *(R)*	659 (62.9)	1		1	
≥50	372 (62.1)	0.97 (0.78-1.19)	0.735	1.00 (0.72-1.39)	0.996
What do you think is your risk of dying from COVID-19 if infected?					
<50% *(R)*	798 (62.5)	1		1	
≥50	233 (63.0)	1.02 (0.80-1.29)	0.879	1.17 (0.87-1.58)	0.297
Perceived risk (total score)					
Low (≤50) *(R)*	549 (63.5)	1		1	
High (>50)	482 (61.7)	0.93 (0.76-1.13)	0.463	0.78 (0.55-1.09)	0.142

^a^ Have been tested or examined by a doctor but negative.

^b^ Have been diagnosed by a doctor.

* Significant at 0.05.

** Significant at 0.001.

In the adjusted analysis, having a healthcare-related job, knowing people in immediate social environment who are or have been infected with SARS-CoV-2, and having been exposed to information about individuals infected with the SARS-CoV-2 on social media or TV were factors associated with good belief in social distancing benefits. Those who were working in healthcare-related sectors had higher odds in believing that social distancing could prevent the SARS-CoV-2 infection compared to those who were working in non-healthcare-related sectors (aOR: 1.39; 95%CI: 1.10-1.77, p=0.007). Respondents who knew individuals in their immediate social environment who were or had been infected with SARS-CoV-2 (either mild cases or serious cases) had a stronger belief in benefits of social distancing (aOR: 1.41; 95%CI: 1.02-1.96) compared to those who did not. In addition, respondents who were exposed to COVID-19 cases from TV or social media (had seen or read about individuals infected) had almost 2.5 times higher odds of belief in the benefits of social distancing compared to those who had never been exposed to COVID-19 cases information (95%CI: 1.60, 3.69) ( Table 2).

### Face mask practices and associated determinants

Out of the total 1,306 respondents, 875 (66.9%) of them were considered to have good face mask practices. The percentage of good practices were varied among countries ranging from 46.1% in Sudan to 88.6% in Chile. Some determinants were identified in univariate analyses such as country, gender, income, religion, sector of workplaces, type of occupation, having COVID-19 comorbidities, knowing people in their immediate social environment who are or have been infected with SARS-CoV-2, exposure to information regarding COVID-19 on social media or TV, and the perceived risk of getting COVID-19 ( Table 3).

**Table 3.** Unadjusted and adjusted logistic regression analyses showing factors associated with good face mask practice ( *n*=1306).

Variable	Good practices *n* (%)	Unadjusted		Adjusted	
		OR (95% CI)	*p*–value	aOR (95% CI)	*p*–value
Country					
Pakistan *(R)*	99 (54.7)	1		1	
Brazil	54 (87.1)	5.59 (2.52-12.42)	<0.001 **	5.68 (2.06-15.66)	0.001 *
Chile	39 (88.6)	6.46 (2.44-17.14)	<0.001 *	8.17 (2.60-25.72)	<0.001 **
Egypt	61 (69.3)	1.87 (1.09-3.21)	0.023 *	1.21 (0.65-2.22)	0.547
India	231 (76.5)	2.70 (1.82-4.00)	<0.001 *	2.47 (1.24-4.94)	0.011 *
Iran	96 (79.3)	3.18 (1.88-5.40)	<0.001 *	2.54 (1.34-4.80)	0.004 *
Nigeria	76 (53.5)	0.95 (0.61-1.48)	0.833	0.50 (0.25-1.00)	0.049 *
Bangladesh	77 (72.0)	2.13 (1.27-3.55)	0.004 *	2.34 (1.29-4.24)	0.005 *
Sudan	65 (46.1)	0.71 (0.46-1.10)	0.126	0.46 (0.26-0.79)	0.005 *
Tunisia	77 (65.3)	1.56 (0.96-2.51)	0.070	1.28 (0.70-2.33)	0.426
Age group (year)					
<20 *(R)*	121 (65.4)	1		1	
21-30	488 (65.6)	1.01 (0.72-1.42)	0.962	0.73 (0.48-1.10)	0.128
31-40	160 (69.3)	1.19 (0.79-1.80)	0.404	0.89 (0.50-1.58)	0.686
41-50	76 (73.8)	1.49 (0.87-2.54)	0.144	1.40 (0.70-2.83)	0.344
>51	30 (69.8)	1.22 (0.60-2.50)	0.586	1.11 (0.46-2.72)	0.812
Gender					
Male *(R)*	362 (63.5)	1		1	
Female	513 (69.7)	1.32 (1.05-1.67)	0.018 *	1.46 (1.11-1.92)	0.007 *
Urbanicity					
Rural *(R)*	163 (64.2)	1		1	
Urban	712 (67.7)	1.17 (0.88-1.56)	0.286	1.03 (0.74-1.44)	0.858
Monthly household income (USD)					
<500 *(R)*	320 (64.6)	1		1	
500-999	149 (66.2)	1.07 (0.77-1.49)	0.681	0.85 (0.59-1.23)	0.391
500-999	112 (74.2)	1.57 (1.04-2.36)	0.030 *	1.06 (0.67-1.67)	0.810
2,000-2,999	76 (70.4)	1.30 (0.83-2.04)	0.257	0.89 (0.53-1.48)	0.646
3,000-4,999	62 (64.6)	1.00 (0.63-1.58)	0.991	0.56 (0.33-0.95)	0.032 *
5,000-7,999	46 (61.3)	0.87 (0.53-1.43)	0.577	0.46 (0.26-0.82)	0.009 *
≥8,000	110 (70.5)	1.31 (0.89-1.93)	0.178	0.76 (0.47-1.25)	0.286
Religion					
Islam *(R)*	464 (62.3)	1		1	
Christian/Protestant/Methodist/Lutheran/Baptist	105 (69.5)	1.38 (0.95-2.02)	0.092	1.71 (0.96-3.04)	0.069
Catholic	62 (71.3)	1.50 (0.92-2.45)	0.102	0.70 (0.34-1.45)	0.339
Hindu	167 (79.1)	2.30 (1.60-3.31)	<0.001 **	1.23 (0.67-2.27)	0.513
Atheist or agnostic	27 (62.8)	1.02 (0.54-1.93)	0.947	0.43 (0.20-0.91)	0.028 *
Others	50 (72.5)	1.59 (0.92-2.76)	0.096	0.70 (0.36-1.36)	0.297
Healthcare related job					
No *(R)*	404 (61.0)	1		1	
Yes	471 (73.1)	1.74 (1.38-2.20)	<0.001 *	1.84 (1.38-2.45)	<0.001 **
Occupation					
Self-employed *(R)*	85 (61.6)	1		1	
Employed for wages	254 (70.6)	1.49 (0.99-2.25)	0.056	1.74 (1.09-2.78)	0.021 *
Out of work for less or more than 1 year	27 (54.0)	0.73 (0.38-1.41)	0.349	0.85 (0.41-1.76)	0.666
Homemaker	14 (58.3)	0.87 (0.36-2.11)	0.762	0.73 (0.28-1.94)	0.532
Student	482 (67.3)	1.28 (0.88-1.87)	0.193	1.63 (1.03-2.60)	0.039 *
Retired or unable to work	13 (72.2)	1.62 (0.55-4.81)	0.384	1.87 (0.54-6.42)	0.321
Have hypertension					
No ^a^ *(R)*	625 (70.0)	1		1	
Yes ^b^	46 (57.5)	0.58 (0.36-0.92)	0.022 *	0.70 (0.39-1.27)	0.245
Do not know	204 (61.3)	0.68 (0.52-0.88)	0.004 *	0.85 (0.57-1.28)	0.444
Have diabetes					
No ^a^ *(R)*	667 (69.3)	1		1	
Yes ^b^	28 (56.0)	0.56 (0.32-1.00)	0.050 *	0.62 (0.30-1.27)	0.190
Do not know	180 (61.2)	0.70 (0.53-0.92)	0.010 *	1.00 (0.65-1.52)	0.982
Have heart disease					
No ^a^ *(R)*	661 (69.2)	1		1	
Yes ^b^	30 (63.8)	0.79 (0.43-1.45)	0.440	1.02 (0.47-2.22)	0.956
Do not know	234 (62.2)	0.73 (0.57-0.95)	0.016 *	1.03 (0.65-1.63)	0.888
Have pulmonary disease					
No ^a^ *(R)*	595 (70.4)	1		1	
Yes ^b^	44 (57.9)	0.58 (0.36-0.93)	0.025 *	0.77 (0.43-1.37)	0.369
Do not know	236 (61.3)	0.67 (0.52-0.86)	0.002 *	0.81 (0.52-1.27)	0.358
Know people in immediate social environment who are or have been infected with COVID-19					
No *(R)*	215 (58.7)	1		1	
Yes	660 (70.2)	1.66 (1.29-2.13)	<0.001 **	1.25 (0.84-1.84)	0.268
Have you seen or read about individuals infected with the COVID-19 on social media or TV?					
No *(R)*	58 (57.4)	1		1	
Yes	817 (67.8)	1.56 (1.03-2.36)	0.034 *	1.69 (1.04-2.75)	0.036 *
What do you think are the chances that you will get COVID-19 in the next month?					
<50% *(R)*	519 (64.9)	1		1	
≥50%	356 (70.4)	1.29 (1.01-1.63)	0.040 *	1.07 (0.72-1.57)	0.748
What do you think is your risk of dying from COVID-19 if infected?					
<50% *(R)*	683 (67.2)	1		1	
≥50%	192 (66.4)	0.97 (0.73-1.28)	0.818	0.99 (0.69-1.42)	0.960
Perceived risk (total score)					
Low (≤50%) *(R)*	427 (65.1)	1		1	
High (>50%)	448 (68.9)	1.19 (0.94-1.50)	0.141	0.97 (0.65-1.44)	0.860

^a^ Have been tested or examined by a doctor but negative.

^b^ Have been diagnosed by a doctor.

* Significant at 0.05.

** Significant at 0.001.

In an adjusted analysis, country, gender, monthly household income, having occupation related to healthcare sectors, types of occupation, and having seen or read about individuals infected with the SARS-CoV-2 on TV or social media were all significantly associated with practicing face mask use ( Table 3). Females had 1.46 times greater odds of having good practices compared to males (95%CI: 1.11-1.92, p=0.007). Compared to those who earned less than $500, respondents who earned $3,000-$4,999 and $5,000-$7,999 had lower odds of face mask-wearing when going out to workplaces or school or other places with aOR: 0.56; 95%CI: 0.33-0.95 and aOR: 0.46; 95%CI: 0.26-0.82, respectively. Those who were working in a healthcare-related job had almost two-fold odds (aOR: 1.84) of having good practice compared to those working in non-healthcare workplaces. Employees working for wages and students also had better face mask use practices compared to those who were self-employed (entrepreneurs). Our data also shows that exposure to coverage of COVID-19 cases in the media was associated with good face mask practices with OR: 1.69 and 95%CI: 1.04-2.75 ( Table 3).

## Discussion

In addition to a massive vaccination campaign, public health measures such as social distancing and face mask use will still continue to play a pivotal role in reducing COVID-19 transmission. Social distancing and face mask use are still highly recommended ^12^ even after COVID-19 vaccination since vaccines cannot fully prevent SARS-CoV-2 infection, ^17^ and vaccines are still difficult to access throughout much of the world. ^18^ Our data suggests that less than 70% of the participants had good beliefs in social distancing (62.6%) and good face mask practices (66.9%).

Our study found that individuals with healthcare-related jobs and who had seen or read about COVID-19 cases on social media or TV had good beliefs in social distancing benefits and face mask practices. In general, healthcare-related workers have better knowledge of COVID-19 than the general population ^19^ and therefore might know better the benefits of public health measures such as social distancing and face mask-wearing in preventing COVID-19. Similarly, people who often get exposed to news related to COVID-19 might also have better knowledge about the disease, ^20^^,^^21^ which might lead to a more positive attitude towards social distancing and better practice of face mask-wearing. A positive association of good knowledge with social distancing and face mask-wearing practices have also been captured in previous investigations. ^22^^,^^23^ This highlights the importance of continuous knowledge dissemination through health campaigns to enhance beliefs in social distancing benefits and mask-wearing among the general population.

Our results suggest that participants who knew people in their immediate social environment who were or had been infected with COVID-19 was significantly associated with good belief in social distancing benefits. Individuals who have had first-hand experience of the pandemic, either by contracting COVID-19 themselves or knowing people who are or have been infected with COVID-19, are more likely to agree and comply with health measures such as social distancing. ^24^ Having friends or relatives who are or have been infected with COVID-19 might increase awareness of serious health consequences of the pandemic ^24^ and increase motivation to protect themselves or the community, resulting in better beliefs and adherence to social distancing recommendations. ^25^ Knowing others with COVID-19, particularly those with a severe course of illness could also impact the perceived risk of disease, which itself could motivate healthy behaviors, as postulated by the Health Belief Model. ^26^

Our study found that females had better face mask practices compared to their male counterparts. This supports previous studies revealing that females had better knowledge on COVID-19 and better practice of preventive measures, e.g., face mask-wearing, compared to men. ^19^^,^^27^^,^^28^ Gender differences in COVID-19 attitudes and behavior have also been observed in another study showing that women were more likely than men to agree and comply with restraining public health policy such as mask-wearing, since they were more likely to perceive COVID-19 as a serious health problem. ^24^ Existing literature also showed that women have better knowledge in emerging infectious diseases, ^29^^,^^30^ and are more favorable to government intervention than men. ^31^^,^^32^ Moreover, women are more likely to be the caregivers of the family, which might cause them to be more worried about getting infected with COVID-19 as they could subsequently transmit the virus to the other family members. ^24^

One interesting finding in our study was that individuals with a higher monthly income had poor face mask-wearing practice. Previous studies reported a positive association between higher income and knowledge on COVID-19 preventive measures ^27^^,^^28^^,^^33^; however, there are contradictory reports on the association between income and face mask practice. ^34^^,^^35^ A study conducted in China reported that the proportion of people wearing a face mask increased linearly with monthly income, ^34^ while another study reported no association between the compliance with face mask-wearing and monthly household income. ^35^ Such findings might be confounded by the fact that face mask-wearing in public places is strictly imposed in some countries, but not in others. ^36^ Moreover, respondents with a higher household income in this study might be more likely to work from home, and thus may not feel the same need to wear face masks as someone who more regularly goes out. Overall, our findings suggest that the government should emphasize public health campaigns targeting men and low-educated people to improve public beliefs and practice of COVID-19 prevention measures, such as social distancing and mask-wearing.

There are some limitations of this study. The use of an online survey excludes some people from lower social-economic classes, those with lower educational attainment, and those who were illiterate. Selection bias might also occur due to the variation in internet access across the countries where the study was conducted. Measurement of mask-wearing practice was based on questions of whether or not participants wore face mask at work/school and grocery stores/food vendors, which might be compulsory in some countries, and may not reflect the real practices of mask-wearing in the community. Participants may also respond in a certain way due to the social desirability bias.

## Conclusion

Our data suggested that there is a substantial percentage of community members in certain low- and middle-income countries who do not believe in social distancing benefits during the COVID-19 pandemic and do not have good face mask-wearing practices. Some determinants associated with negative beliefs on the benefits of social distancing and poor face mask practices have been identified through this study, and these could be used by the governments or other organizations to increase adherence to social distancing and face mask-wearing practices in the community.

## Data availability

### Underlying data

Figshare: 'Beliefs on social distancing and face mask practices during the COVID-19 pandemic in low- and middle-income countries: a cross-sectional study'. https://doi.org/10.6084/m9.figshare.19105238 ^37^

This project contains the following underlying data:

- Master Table.xlsx [Table containing the raw data of the study]

### Extended data

This project contains the following extended data:

- Study Questionnaire.pdf

### Reporting guidelines

‐ STROBE_Checklist.pdf. STROBE checklist for 'Beliefs on social distancing and face mask practices during the COVID-19 pandemic in low- and middle-income countries: a cross-sectional study'. https://doi.org/10.6084/m9.figshare.19105238 ^37^

Data are available under the terms of the Creative Commons Attribution 4.0 International license (CC-BY 4.0).
