# Supplementary material for: Beliefs on social distancing and face mask practices during the COVID-19 pandemic in low- and middle-income countries: a cross-sectional study
Source: F1000Res. 2026 Mar 5;11:206. Originally published 2022 Feb 17. [Version 2] doi: 10.12688/f1000research.79534.2 (PMC12989056; doi:10.12688/f1000research.79534.2)
Supplement: Peer review history

# Reviewer report: Reviewer response for version 2

**DOI:** 10.5256/f1000research.197181.r464970

**Published:** 2026-03-17

**Authors:** Yousef Khader

**Affiliations:** 1 Department of Public Health, Community Medicine and Family Medicine, Faculty of Medicine, Jordan University of Science and Technology, Irbid, Jordan

This manuscript addresses an important public health topic by examining beliefs in social distancing and face mask practices during the COVID-19 pandemic across multiple low- and middle-income countries. The introduction clearly establishes the relevance of preventive behaviors in controlling SARS-CoV-2 transmission and provides a solid rationale for the study. The methods are well described, with a transparent explanation of the cross-sectional design, survey instrument, and analytical approach. The inclusion of respondents from ten countries strengthens the contribution of the study by providing comparative insights across diverse settings. The results are presented clearly and highlight meaningful determinants of preventive behaviors, while the discussion appropriately interprets the findings in relation to existing literature and acknowledges key limitations. Overall, the study provides useful evidence that can help inform targeted public health communication strategies aimed at improving adherence to non-pharmaceutical interventions during infectious disease outbreaks.

Is the work clearly and accurately presented and does it cite the current literature?

Yes

If applicable, is the statistical analysis and its interpretation appropriate?

Yes

Are all the source data underlying the results available to ensure full reproducibility?

Yes

Is the study design appropriate and is the work technically sound?

Yes

Are the conclusions drawn adequately supported by the results?

Yes

Are sufficient details of methods and analysis provided to allow replication by others?

Yes

Reviewer Expertise:

Epidemiology

I confirm that I have read this submission and believe that I have an appropriate level of expertise to confirm that it is of an acceptable scientific standard.

# Reviewer report: Reviewer response for version 2

**DOI:** 10.5256/f1000research.197181.r464971

**Published:** 2026-03-14

**Authors:** Roland Nnaemeka Okoro

**Affiliations:** 1 Department of Clinical Pharmacy and Pharmacy Administration, University of Maiduguri, Maiduguri, Borno, Nigeria

My comments were satisfactorily addressed

Is the work clearly and accurately presented and does it cite the current literature?

Yes

If applicable, is the statistical analysis and its interpretation appropriate?

Partly

Are all the source data underlying the results available to ensure full reproducibility?

Yes

Is the study design appropriate and is the work technically sound?

Yes

Are the conclusions drawn adequately supported by the results?

Yes

Are sufficient details of methods and analysis provided to allow replication by others?

No

Reviewer Expertise:

Pharmacy Education and Pharmacy Practice, Tropical Diseases with emphasis on Malaria, and Pharmacotherapeutics

I confirm that I have read this submission and believe that I have an appropriate level of expertise to confirm that it is of an acceptable scientific standard.

# Reviewer report: Reviewer response for version 1

**DOI:** 10.5256/f1000research.83528.r290573

**Published:** 2024-07-22

**Authors:** Roland Nnaemeka Okoro

**Affiliations:** 1 Department of Clinical Pharmacy and Pharmacy Administration, University of Maiduguri, Maiduguri, Borno, Nigeria

The study is interesting and a show of a good teamwork, although I have identified some issues that need to be addressed by the authors.

**Introduction:**

 Paragraph 1: As far as COVID-19 is concerned, it is not just “regular handwashing” but “regular handwashing with soap under running water”

**Methods:**

 Paragraph 1: What informed the choice of counties selected for the study?

 Paragraph 2: The minimum sample size calculated using the provided variables should be stated.

 Paragraph 3:

- Brief description of the study instrument should stand alone before data collection. Information on the study variables should be part of study instrument description. Information on how this instrument was validated is required.
- How was cut-off of 50% used to categorize the perceived risk determined?
- Data collection should be stated thereafter. The duration of data collection should be stated and how data collection was stopped in each country that was included in the study.
- Paragraph 5: Nothing was mentioned about how the results were presented. All that was stated here was how factors were determined.

**Results:**

- Overall, how many people were invited to participate in the study? What was the response rate?
- Authors stated that 203 respondents were exclude due to incomplete data, but this was not capture as an exclusion criterion in the method section.

**Discussion:**

 Well written

**Conclusion:**

 Well written

Is the work clearly and accurately presented and does it cite the current literature?

Yes

If applicable, is the statistical analysis and its interpretation appropriate?

Partly

Are all the source data underlying the results available to ensure full reproducibility?

Yes

Is the study design appropriate and is the work technically sound?

Yes

Are the conclusions drawn adequately supported by the results?

Yes

Are sufficient details of methods and analysis provided to allow replication by others?

No

Reviewer Expertise:

Pharmacy Education and Pharmacy Practice, Tropical Diseases with emphasis on Malaria, and Pharmacotherapeutics

I confirm that I have read this submission and believe that I have an appropriate level of expertise to confirm that it is of an acceptable scientific standard, however I have significant reservations, as outlined above.

# Response

**Published:** 2026-02-26

**Authors:** Harapan Harapan

**Affiliations:** Syiah Kuala University, Indonesia

Dear Reviewer,

 Thank you for your constructive comments and suggestions on the first version of our article. We have revised the manuscript accordingly. Detailed point-by-point responses to each of your comments are provided below.

**REVIEWER COMMENT:**

 The study is interesting and a show of a good teamwork, although I have identified some issues that need to be addressed by the authors.

**Introduction:** Paragraph 1: As far as COVID-19 is concerned, it is not just “regular handwashing” but “regular handwashing with soap under running water”

**AUTHORS RESPONSE:**

 Thank you for this correction. We have revised the wording in the **Introduction** accordingly.

**ADDED/REVISED TEXT:**

 Replace: “regular handwashing”

 With: “regular handwashing with soap under running water”

**REVIEWER COMMENT:**

 Methods - Paragraph 1: What informed the choice of counties selected for the study?

**AUTHORS RESPONSE:**

 Thank you. We have now clarified the rationale for country selection in the **Study design and setting** section, including feasibility of online survey implementation, availability of local collaborators, and representation of LMICs across multiple regions.

**ADDED/REVISED TEXT:**

**Methods – Study design and setting**

 “The participating countries were selected based on the feasibility of online survey implementation, availability of local collaborators in each country to support survey dissemination, and the intention to include LMIC settings from multiple geographic regions.”

**REVIEWER COMMENT:**

 Methods - Paragraph 2: The minimum sample size calculated using the provided variables should be stated.

**AUTHORS RESPONSE:**

 Thank you. We have now explicitly stated the minimum sample size and how it was calculated in the **Study population and sampling** section.

**ADDED/REVISED TEXT:**

**Methods – Study population and sampling**

 “The minimum sample size for estimating a proportion was calculated using a 5% margin of error, a 95% confidence level and a 50% conservative estimate of respondents having good beliefs in social distancing benefits and face mask practices, yielding a minimum required sample size of 385 participants.”

**REVIEWER COMMENT:**

 Methods - Paragraph 3: Brief description of the study instrument should stand alone before data collection. Information on the study variables should be part of study instrument description. Information on how this instrument was validated is required.

**AUTHORS RESPONSE:**

 Thank you for this helpful suggestion. We restructured the **Methods** section to separate the **Study instrument** description from the **Data collection** subsection. We also added a clearer description of the questionnaire domains and a statement on instrument review/pretesting before implementation. Study variables section is now moved under directly under **Study instrument**.

**ADDED/REVISED TEXT:**

**Methods – Study instrument** *(new subsection before Data collection)*

**“Study instrument”**

 A structured questionnaire was developed to assess respondents’ demographic characteristics, belief in the benefits of social distancing, face mask practices, COVID-19-related exposures, comorbidities, and perceived risk toward COVID-19. The questionnaire included closed-ended items and Likert-type response options for the belief domain. A copy of the survey can be found under Extended data. ^37^ Before implementation, the questionnaire was reviewed by experts in the fields of virology and public health as well as the country collaborators for clarity and relevance. The questions within the questionnaire were tested and validity was confirmed prior to being used in the study. Wording and sequence were refined based on the validity assessment prior to data collection.

**REVIEWER COMMENT:**

 Methods - Paragraph 3: How was cut-off of 50% used to categorize the perceived risk determined?

**AUTHORS RESPONSE:**

 Thank you. We clarified in the **Study variables** section that the 50% cut-off was used as a pragmatic midpoint threshold to dichotomize perceived risk scores for interpretation and analysis, rather than a universally validated cut-off.

**ADDED/REVISED TEXT:**

**Methods – Explanatory variables**

 “For analysis, responses were dichotomized using a pragmatic midpoint threshold (≤50% vs. >50%) to facilitate interpretation. In addition, the two scores from summed (ranges: 0–200) and respondents who achieved more than 50% of the total score (i.e., 100 scores out of 200) were classified as having high perceived risk; conversely, those with less than 50% were classified as having a low perceived risk.”

**REVIEWER COMMENT:**

 Methods - Paragraph 3: Data collection should be stated thereafter. The duration of data collection should be stated and how data collection was stopped in each country that was included in the study.

**AUTHORS RESPONSE:**

 Thank you. We revised the **Data collection** subsection to clearly state the data collection period (February to May 2021), the dissemination process across countries, and how the online survey was closed at the end of the study period.

**ADDED/REVISED TEXT:**

**Methods – Data collection**

 “Data collection was conducted from February to May 2021 for all countries. The survey remained open during this period and was closed at the end of the data collection window, after which no further responses were accepted.”

**REVIEWER COMMENT:**

 Methods - Paragraph 5: Nothing was mentioned about how the results were presented. All that was stated here was how factors were determined.

**AUTHORS RESPONSE:**

 Thank you. We have revised the **Statistical analysis** section to clearly state how the results were summarized and presented, in addition to how associated factors were analyzed.

**ADDED/REVISED TEXT:**

**Methods – Statistical analysis**

 “The responses were downloaded from SurveyMonkey into Microsoft Excel, cleaned, coded, and then exported to the Statistical Package for Social Sciences (SPSS) for analysis. Descriptive statistics for respondents’ sociodemographic characteristics, beliefs in the benefits of social distancing, and face mask practices were summarized and presented as frequencies and percentages. The numbers and proportions of respondents with good belief in the benefits of social distancing and good face mask practice were calculated.”

**REVIEWER COMMENT:**

 Results - Overall, how many people were invited to participate in the study? What was the response rate?

**AUTHORS RESPONSE:**

 Thank you. We clarified in the **Results** that because the survey was disseminated through open online channels and snowball sharing, the number of individuals who received/viewed the invitation could not be determined; therefore, a conventional response rate could not be calculated.

**ADDED/REVISED TEXT:**

**Results – Demographic characteristics (opening paragraph)**

 “During the study period, 1,849 responses were received. Because the survey was disseminated through open online channels and social sharing, the total number of individuals who received or viewed the invitation could not be determined; therefore, a conventional response rate could not be calculated. In this present study, a total of 203 respondents were excluded due to incomplete data, leaving 1,646 respondents for the analysis of belief in social distancing benefits, where more than 20% of them were from India.”

**REVIEWER COMMENT:**

 Results - Authors stated that 203 respondents were exclude due to incomplete data, but this was not capture as an exclusion criterion in the method section.

**AUTHORS RESPONSE:**

 Thank you for identifying this inconsistency. We have revised the **Methods (Study population and sampling)** section to explicitly state that responses with incomplete data on variables required for analysis were excluded.

**ADDED/REVISED TEXT**:

**Methods – Study population and sampling**

 “Responses with incomplete data for variables required in the analyses were also excluded.”

**REVIEWER COMMENT:**

 Discussion - Well written

 Conclusion - Well written

**AUTHORS RESPONSE**: Thank you.

**ADDED/REVISED TEXT**: NA

# Reviewer report: Reviewer response for version 1

**DOI:** 10.5256/f1000research.83528.r141134

**Published:** 2022-06-27

**Authors:** Yousef Khader

**Affiliations:** 1 Department of Public Health, Community Medicine and Family Medicine, Faculty of Medicine, Jordan University of Science and Technology, Irbid, Jordan

This study interesting study was conducted to assess the level of belief in social distancing and face mask practices in communities in low- and middle-income countries (LMICs) and to identify their possible determinants. The manuscript is well written.

 The following comments need to be addressed by authors:

1. Please show the calculated sample size. Is it calculated for each country? You should show the sample size calculation considering the logistic regression analysis and not only for estimating a single proportion. Please elaborate on how you recruited people from each country. Is there a focal person in each country to reach people in each country?
2. Please mention the limitations that are associated with the sampling approach you used?  
3. The 80% cut-off: Is it possible to cite a reference that is related to the establishing this cut-off value for any standard questionnaire?
4. Did you consider clustering within country in the analysis (Multilevel analysis)?

Is the work clearly and accurately presented and does it cite the current literature?

Yes

If applicable, is the statistical analysis and its interpretation appropriate?

Yes

Are all the source data underlying the results available to ensure full reproducibility?

Yes

Is the study design appropriate and is the work technically sound?

Yes

Are the conclusions drawn adequately supported by the results?

Yes

Are sufficient details of methods and analysis provided to allow replication by others?

Yes

Reviewer Expertise:

Epidemiology

I confirm that I have read this submission and believe that I have an appropriate level of expertise to confirm that it is of an acceptable scientific standard.

# Response

**Published:** 2026-02-26

**Authors:** Harapan Harapan

**Affiliations:** Syiah Kuala University, Indonesia

Dear Reviewer,

 Thank you for your constructive comments and suggestions on the first version of our article. We have revised the manuscript accordingly. Detailed point-by-point responses to each of your comments are provided below.

**REVIEWER COMMENT:**

 This study interesting study was conducted to assess the level of belief in social distancing and face mask practices in communities in low- and middle-income countries (LMICs) and to identify their possible determinants. The manuscript is well written.

**AUTHORS RESPONSE:**

 Thank you for your constructive and encouraging comments. Point-by-point responses to each of your comments are provided below.

**REVIEWER COMMENT:**

 The following comments need to be addressed by authors:

 1. Please show the calculated sample size. Is it calculated for each country? You should show the sample size calculation considering the logistic regression analysis and not only for estimating a single proportion. Please elaborate on how you recruited people from each country. Is there a focal person in each country to reach people in each country?

**AUTHORS RESPONSE:**

 Thank you for this important comment. We have revised the **Methods** section to explicitly report the minimum sample size calculation (95% confidence level, 5% margin of error, and 50% expected proportion), and clarified that this estimate was used as a minimum overall sample size, not a country-specific sample size. We also clarified that the final sample exceeded this minimum to support multivariable logistic regression and country-level comparisons. In addition, we expanded the recruitment procedure to explain that the survey link was distributed via social media and networks across participating countries, and that local collaborators/co-authors served as focal persons in each country to facilitate dissemination.

**ADDED/REVISED TEXT**:

**Methods – Study population and sampling**

 “The minimum sample size for estimating a proportion was calculated using a 5% margin of error, a 95% confidence level and a 50% conservative estimate of respondents having good beliefs in social distancing benefits and face mask practices, yielding a minimum required sample size of 385 participants. This calculation was applied as the minimum overall sample size for the study; not for each country separately. Recruitment was continued beyond this minimum to improve precision and support multivariable logistic regression analyses and country-level comparisons.”

**Methods – Data collection**

 “In each participating country, local collaborators/co-authors served as focal persons to facilitate dissemination of the survey link through their professional and community networks.”

**REVIEWER COMMENT:**

 2. Please mention the limitations that are associated with the sampling approach you used?

**AUTHORS RESPONSE:**

 Thank you. Thank you for this important comment. We have revised the limitations paragraph in the Discussion to explicitly acknowledge the limitation related to the sampling approach. Specifically, we added that the use of a non-probability convenience sampling approach (including online and snowball dissemination) may have introduced selection bias and limited the generalizability of the findings to the broader population in each participating country.

**ADDED TEXT**:

**Discussion – Limitations**

 “In addition, the use of a non-probability convenience sampling approach (including online and snowball dissemination) may have introduced selection bias and limited the generalizability of the findings to the broader population in each participating country.”

**REVIEWER COMMENT:**

 3. The 80% cut-off: Is it possible to cite a reference that is related to the establishing this cut-off value for any standard questionnaire?

**AUTHORS RESPONSE:**

 Thank you for this important comment. We have revised the relevant sentence in the **Study variables **to clarify that the 80% cut-off was used as an operational (pragmatic) threshold, consistent with its use in previous studies cited in our manuscript [15,16], and not as a universally standardized cut-off for this questionnaire.

**ADDED/REVISED TEXT:**

**Methods – Study variables (social distancing belief)**

 The 80% cut-off was used as an operational (pragmatic) threshold to dichotomize the variable into good and poor categories, consistent with its use in previous studies, ^15 ,16^  rather than as a universally standardized cut-off for this questionnaire.

**REVIEWER COMMENT:**

 4. **Did you consider clustering within country in the analysis (Multilevel analysis)?**

**AUTHORS RESPONSE:**

 Thank you for raising this point. We have clarified in the **Statistical analysis** section that multilevel (hierarchical) modeling was not performed in the current analysis and that country was included as an explanatory variable in the regression models. We also added this as a limitation in the **Discussion**.

**ADDED/REVISED TEXT:**

**Methods – Statistical analysis**

 “Multilevel modeling to account for clustering by country was not performed in the present analysis; instead, country was included as an explanatory variable in the regression models.”

**Discussion – Limitations**

 “Furthermore, in this study, multilevel modelling was not performed to explicitly account for within-country clustering. Although country was included as a covariate in the regression models, residual intra-country correlation may still have remained.”
